# A Natural Hybrid Zone of Swordtails Reveals Molecular Insights Into the Adaptive Genomic Basis of Thermal Tolerance

**DOI:** 10.1111/mec.17584

**Published:** 2024-11-14

**Authors:** Carina M. Lai, Brenna C. M. Stanford, Sean M. Rogers

**Affiliations:** ^1^ Department of Biological Sciences University of Calgary Calgary Alberta Canada; ^2^ Bamfield Marine Sciences Centre Bamfield British Columbia Canada

Environmental stressors influence phenotypic variation at all biological levels—from molecular and tissue specific mechanisms to whole organism performance—necessitating highly integrative approaches when studying the evolution of organism responses to the environment. For example, temperature structures patterns of biodiversity across the globe and is pivotal in shaping physiological, behavioural and morphological traits. Indeed, as an ‘abiotic master factor’ (Sunday, Bates, and Dulvy 2012; Walther et al. 2002), temperature can induce changes in the expression of thousands of genes and a plethora of biological pathways (Bhardwaj et al. 2015; Long et al. 2013; Stanford et al. 2020). While thermotolerance has been identified as the fastest evolving trait in nature (Barrett et al. 2011), determining its genomic architecture has been undeniably challenging. Now, in this issue of Molecular Ecology, Payne et al. (2022) adopt an integrative approach to understand the molecular mechanisms that contribute to variation in thermotolerance using a powerful natural system of two species of swordtail fish endemic to eastern Mexico and their hybrids. By combining QTL mapping, gene expression, population ancestry and thermotolerance data in experimental and natural populations, the authors uncover evidence of extensive genetic incompatibilities leading to a reduction in hybrid thermotolerance. Payne et al.'s findings highlight the highly polygenic and modular nature of thermotolerance and the potential negative consequences of hybridisation for the performance of ecological traits.

For their study, the authors used two parental species, *Xiphophorus malinche* and *X. birchmanni*, that occur at high and low elevation streams, respectively, and exhibit natural variation in thermotolerance. Hybridisation occurs at intermediate elevations where parental ranges overlap, resulting in natural hybrid zones with varying degrees of hybrid ancestry and intermediate thermotolerance (Figure 1.). Hybrid zones have long been considered natural windows into the genetics of adaptation and evolution of speciation (Harrison 1993). Due to incomplete reproductive isolation, recombination in hybrid crosses can unveil a wide multitude of novel genotypes that have yet to be removed by natural selection. Hybrid zones thus offer valuable insights when characterising the genetic basis of ecological traits and their underlying adaptive mechanisms.

**FIGURE 1:**
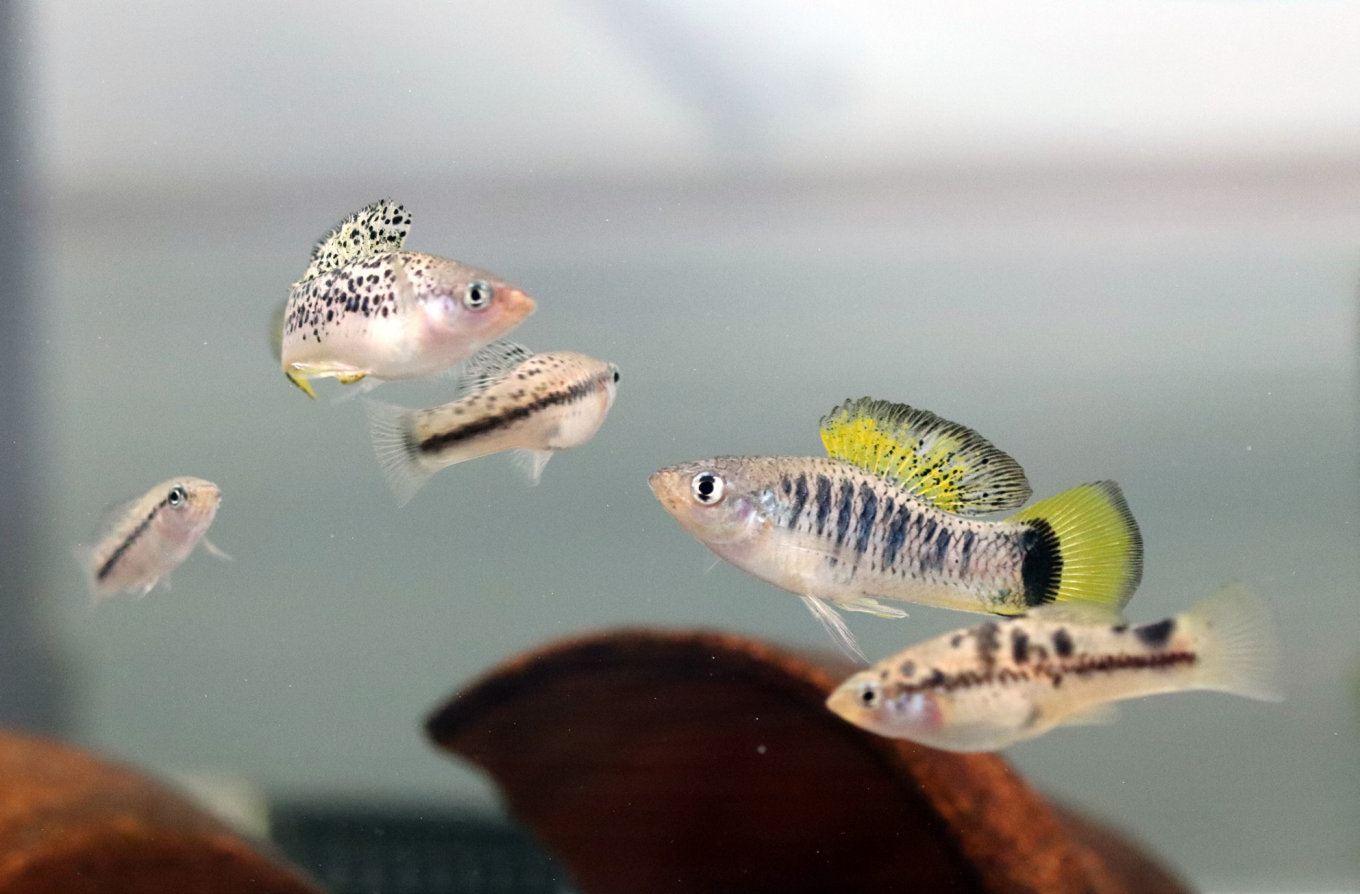
Male and female *Xiphophorus malinche–X. birchmanni* hybrids from the natural Tlatemaco hybrid population located in eastern Mexico (Photo credit: Daniel L. Powell).

Payne et al. (2022) leverage their study system using both artificial crosses (from wild‐caught pure parental individuals) and natural hybrid populations to test how hybridisation has influenced the evolution of thermotolerance, and apply a broad range of genomic methods to link gene regulation with biological phenotypes associated with thermal responses. They first use a quantitative trait locus (QTL) mapping approach with laboratory crosses to identify genomic regions associated with critical thermal maximum (CT_max_), discovering a significant QTL on chromosome 22. A second putative interacting QTL was also identified on chromosome 15 following a second QTL scan including genotypes at the chromosome 22 peak as an interaction. Rather than be associated with differences in CT_max_ between the parental species, however, individuals with heterozygous ancestry at the chromosome 22 QTL had a lower average CT_max_, indicating loci contributing towards genetic incompatibilities in hybrids that exhibit underdominance, or ‘heterozygote disadvantage’. Such results are compelling as underdominant alleles can persist in hybrid populations over extended time periods and contribute to the maintenance of hybrid zones due to a balance between dispersal and selection against unfit hybrid genotypes (Hewitt 1988; Gilbert, Moinet, and Peischl 2022). Barriers to gene flow in turn promote divergence between parental populations and can reinforce the evolution of reproductive isolation and speciation (Butlin and Smadja 2018). Additionally, these findings highlight the costs of hybridisation and the implications of such costs for adaptive introgression, which has been proposed to facilitate rapid adaptation via the transmission of beneficial alleles between hybridising species (Hedrick 2013). If hybrid incompatibilities exist elsewhere in the genome, however, any fitness benefits conferred by introgressed alleles will be offset by negative interactions associated with unfavourable underdominant genotypes.

To further elucidate the role of gene regulation in thermotolerance and the potential of misregulation of thermotolerance genes and its effects in hybrids, Payne et al. (2022) examined differences in gene expression between parental species and hybrids. Using RNA‐sequencing, they quantified gene expression throughout the transcriptome in laboratory‐reared *X. malinche* , *X. birchmanni* and F_1_ hybrid individuals in response to a control or thermal stress treatment. The authors observed a considerable level of misexpression in F_1_ hybrids in response to thermal stress, meaning these genes, as predicted by theory, had significantly higher or lower expression levels compared to either parental species in these treatments. Specifically, 9% and 3% of thermally responsive genes in the parental species were misexpressed in at least one treatment of F_1_ brains and livers, respectively, suggesting that gene interactions contributed to widespread hybrid incompatibilities throughout the genome. This included the misexpression of four genes in the chromosome 22 QTL region, suggesting that genetic incompatibilities at these loci may explain the observed underdominance at this QTL.

The authors also conducted pathway analyses, which aim to group genes that behave in a coordinated fashion to a factor of interest. Such analyses contribute towards organising large gene expression datasets into biologically relevant groups and pathways, providing more nuanced understandings of both the organism‐level effects of modified gene expression and the targets of adaptive divergence (e.g., see Stanford et al. 2020). The authors tested the biological pathways that were most disrupted by misexpression in artificial hybrids in response to temperature using weighted gene co‐expression network analysis (WGCNA) (Langfelder and Horvath 2008). Notably, one gene group from the WGCNA that exhibited a significant correlation with temperature treatment in both brain and liver tissues included circadian clock genes. Changes to the regulation of circadian rhythm with temperature has been found to be conserved across taxa and likely plays a fundamental role in the thermal stress response (Glaser and Stanewsky 2005; Sua‐Cespedes et al. 2021). In this case, Payne et al. (2022) show that several clock genes were misexpressed in response to temperature in F_1_ hybrids. This pattern was largely driven by clock genes that were responsive to thermal stress in the parental species, but failed to show a response in hybrids, suggesting that disruptions to the regulation of circadian function could negatively impact hybrid thermotolerance. These results demonstrate how hybrid incompatibilities can deleteriously affect fundamental biological functions and homeostasis, which may have corresponding ecological consequences for hybridisation dynamics in natural populations.

Payne et al. (2022) went back to the natural hybrid zone to examine patterns of ancestry and test predictions that molecular divergence at these regions harbouring hybrid incompatibilities were being maintained by selection. Several genes under the chromosome 22 and chromosome 15 QTLs had higher than average *X. birchmanni* ancestry in both hybrid populations compared to that of the genomic background, as well as two clock genes demonstrated to be misexpressed in F_1_ hybrids. This suggests that alleles that are prone to misexpression in hybrids may be selected against in the wild due to their deleterious fitness consequences.

It is increasingly recognised in molecular ecology that the integration of multiple approaches is necessary to understand the genomic variation underlying responses to environmental stress. Payne et al. (2022) combine QTL mapping, differential expression, pathway analyses and population ancestry analyses to uncover the complex mechanisms underlying reduced thermotolerance in *X. malinche* and *X. birchmanni* hybrids. Their work complements other recent studies (Bugg et al. 2023; Popovic and Riginos 2020) that reinforce the necessity of experimental gene expression analyses to study the evolution of thermotolerance in natural populations. In this case, the successful integration of the ecology of gene expression is particularly novel. While extensive lists of differentially expressed genes in studies of adaptive divergence have become common, this study employs both experimental and ecological approaches that link variation at the molecular level to observable phenotypic variation. Such studies aiming to elucidate the genomic basis of complex phenotypes are necessary for our understanding of gene functions and their role in adaptation and evolution. Nevertheless, testing of more phenotypes related to thermotolerance and other ecological traits, at all levels of biological organisation, is necessary if we are to accurately characterise molecular variation in the context of ecological function (Pavey et al. 2012). Follow‐up experiments would benefit from establishing the function of candidate loci using innovative gene editing technologies (Bono, Olesnicky, and Matzkin 2015). Here, in addition to conducting a comprehensive investigation into the mechanisms that underlie hybrid incompatibilities, Payne et al. (2022) present an elegant example of how the integration of genomic and transcriptomic tools, when applied to ecological questions, may lead to novel insights on the molecular forces driving evolutionary processes.

## Author Contributions

C.M.L., B.C.M.S., and S.M.R. wrote and edited the perspective.

## Conflicts of Interest

The authors declare no conflicts of interest.
